# Combined use of clozapine and cariprazine in treatment-resistant schizophrenia, is it a good choice?

**DOI:** 10.1192/j.eurpsy.2021.2111

**Published:** 2021-08-13

**Authors:** H. Becerra Darriba

**Affiliations:** Centro Salud Mental De Tudela - Hospital Reina Sofía, Servicio Navarro de Salud - Osasunbidea., Tudela (Navarra), Spain

**Keywords:** Cariprazine, treatment-resistant schizophrenia, clozapine

## Abstract

**Introduction:**

Treatment-resistant schizophrenia (TRS) affects 30% of people with a diagnosis of schizophrenia, and is defined as nonresponse to at least two trials of antipsychotic medication of adequate dose and duration. Clozapine is the only evidence-based treatment for TRS. Cariprazine may be considered significantly more efficacious than risperidone in improving negative symptoms of schizophrenia.

**Objectives:**

To describe the experience of using cariprazine in combination with clozapine in patients with refractory schizophrenia and negative symptoms.

**Methods:**

Qualitative design. We present a case report study of a 47-year-old male with a diagnosis of TRS, treated in our outpatient mental health clinic for twenty years. The patient experiences crystallized delusional ideas of harm, self-referential, paranoid and mystical-messianic content, phenomena of theft and thought reading, egodistonic auditory hallucinations. No substance use disorder was observed. He made several suicide attempts in the context of intense suffering and psychotic anguish. Clozapine 400mg/day was instituted after no response to treatment with amisulpride, paliperidone, olanzapine or aripiprazole. The intensity of positive symptoms was reduced (experiences of damage, commenting and insulting auditory hallucinations, self-referentiality), as well as the emotional and behavioral repercussions. Persistent negative symptoms appeared such as apathy, abulia, clinophilia, anergy, social isolation, affective flattening, impairing his functionality.

**Results:**

Neuroimaging and periodic blood tests results were normal. Oral cariprazine was added in ascending doses up to 4.5mg with good tolerance. The patient showed remission of apathy, enhancement of behavioral activation, socialization and motivation to perform occupational activities.

**Conclusions:**

Combinations of clozapine with partial agonists may improve the quality of life in refractory schizophrenia.

**Disclosure:**

No significant relationships.

